# Miscellany

**Published:** 1911-08

**Authors:** 


### MISCELLANY

---

THE Buffalo General Hospital, having instituted an automobile-ambulance, has generously donated its old horse-drawn vehicle to the hospital association of the Tonawandas.

---

THE American Institute of Homoeopathy, at its annual meeting June 26-30, elected Dr. Thomas H. Carmichael of Philadelphia, president; Dr. William H. Dieffenbach of New York and Dr. Clara E. Gary of Boston, vice-presidents.

---

DR. Thomas S. Crowe of Chicago, official physician for Cook County, advocates legislation requiring landlords to furnish fly screens for all doors and windows. The hygienic value of screens needs no discussion but it may not be out of place to point out that the tendency, in this country, to impose not only most of the direct taxation but all sorts of obligations and responsibilities upon property owners, instead of upon tenants, creates irresponsibility on the one hand, and, on the other, gradually confiscates capital by making it unproductive and thus discourages home ownership. This fact should be borne in mind in all legislation regarding buildings.

---

IF we give an individual with an unbroken bronchial mucosa the salicylates and then test the secretion we obtain from the bronchi for salicylic acid, we get no r[illegible]on. But when the mucosa is broken or an inflammatory ex[illegible]te is present, salicylic acid is found in the sputum, as in pulmonary tuberculosis, pulmonary abscess and gangrene, and the like.

The test is performed as follows: 30 gr. of sodium salicylate (pure natural) are given in ten grain doses after meals and the sputum collected 12 to 15 hours later. The sputum is acidulated and shaken with 95 per cent. alcohol to precipitate the albumins and mucin, which do not carry any of the salicylates. Filter out this precipitate and alkalinise the filtrate and evaporate. Redissolve the residue in slightly acidulated water and add lead acetate. This causes another precipitate to form, which is again taken out by filtration. This filtrate is then extracted with ether. The ethereal extract is evaporated and tested by adding about ten c.c. of water and one c.c. of a 10 per cent. solution of ferric chloride. A violet color denotes a positive reaction.

---

MILK of Bismuth is manufactured by the well known pharmaceutical house of Eli Lilly & Company, Indianapolis. It is an antacid, astringent, antiseptic and gastric sedative.

Each fluid ounce holds in simple suspension, in distilled water, the bismuth equivalent of 20 grains bismuth subnitrate. It is free from sugar, gums, preservatives and other impurities.

The value of the insoluble bismuth combinations on inflamed and irritated mucous surfaces lies in their coating power, giving mechanical protection. The coating power increases with the fineness of subdivision and the lighter and bulkier the bismuth precipitate, the finer the subdivision and the greater the coating power. For this reason the precipitate in Milk of Bismuth, Lilly, is made very light and bulky, consequently each fluid ounce is equivalent to only 20 grains of subnitrate; at the same time its coating power is greater and more efficient than the usual preparations which contain 40 grains.

Milk of Bismuth, Lilly, agrees with sensitive stomachs and will be found very effective in the treatment of acute, subacute and chronic gastritis, intestinal inflammation, diarrheas, dysentery and similar disorders.

It is supplied by the drug trade in one pint, five pint and one gallon bottles.

---

ACTING Census Director Falkner has received from Dr. J. A. Hill, chief statistician for revision and results, in the census bureau, a preliminary count of the population in institutions comprising prisons, institutions for juvenile delinquents, almshouses and institutions for the insane and feeble-minded. The enumeration includes the number present in the institutions on January 1, 1910, and the number admitted and discharged during the year 1910. A few institutions still remain to be heard from.

According to this preliminary count the prison population on January 1, 1910, was 109,311; the admissions or commitments to prison during the year 1910 were 462,530; and the number of prisoners discharged during that year on account of expiration of sentence, or other reasons, including also deaths, was 458,996.

The last previous census of prisoners was taken June 30, 1904, and at that time the prison population was 81,772, and the admissions or commitments during that year 149,691. These figures, however, are not comparable with those for the year 1910, for the reason that the 1910 enumeration included cases of imprisonment for nonpayment of fine, while the census in 1904 did not include such cases.

Accordingly, the marked increase in prison population, and especially in commitments, does not reflect an increase in crime but is largely accounted for by this difference in the scope of the two censuses.

The census bureau will be able later to segregate from the 1910 figures the cases of imprisonment for nonpayment of fine and thereby obtain a figure which will be fairly comparable with the enumeration of six years ago. The larger number of admissions reported, as compared with the population present on January 1, is indicative of the fact that a large proportion of the commitments are for short sentences and for minor offenses. In the final census report the prisoners will be classified with reference to the offense for which sentenced and the term of sentence imposed.

The number of juvenile delinquents reported at the Census of 1910 in institutions for that class was 22,903. This differs but little from the number reported in 1904, which was 23,034.

The number of paupers in almshouses on January 1, 1910, was 83,944. The number admitted during the year 1910 was 106,457, and the number discharged or dying during that year was 100,858. In 1904 the pauper population was 81,764 at the beginning of the year; the admissions during the year were 81,412; and the discharges, or deaths, 77,886.

The enumeration of the insane in asylums indicates a very striking increase in this class of the population. In 1904 the number of insane in institutions was 150,151. In 1910 this number had increased to 184,123, an increase of 22.6 per cent. in six years. The number of commitments to insane asylums during the year 1904 was 49,622, and during the year 1910 was 59,628, an increase of 20.2 per cent.

In 1904 the feeble minded in institutions numbered 14,347 in 1910 the number was 20,199. The number of commitments to institutions for this class increased from 2,599 in 1904 to 3,848 in 1910.

DR. JOHN W. DRAPER (*Indianapolis Medical Journal*), made the first photographic likeness of any person in the country. The *Literary Digest* for May 13, says that the model for the first portrait which was taken in 1839, was Dr. Draper's sister and his substitute for the modern camera was an old cigar box with a spectacle lens. The subject had the pretty name of Dorothy Catherine Draper. Jacques Daguerre invented the process that bears his name. Daguerreotypes are now simply mementoes.

---

OHIO has recently strengthened its building laws by placing their inforcement in the hands of the State Fire Marshal, the Inspector of workshops and factories and the State Board of Health. A novel feature is the requirement for public buildings, of a plant capable of furnishing a uniform temperature of 65 degrees Fahrenheit, and of a ventilating system which shall change the air at least six times an hour. The new code applies not only to new buildings but to old ones when undergoing repairs.

---

### Plain Truths About Advertisements

IT costs about a cent and a half to reach a prospective customer by mail—two cents and a half if the envelope is sealed. It costs, averaging MEDICAL JOURNAL rates and circulation, from a quarter to half a cent to put before the customer as much space as a postal card and about half a cent to a cent to send him as long a message as it would be worth while to print, providing the mesage is sent through the columns of a medical journal.

It does not, as a rule, pay to try to get personal attention for an advertisement by passing it off as a personal communication at letter rates. If you get a man to listen to your business proposition by explicit or implicit misrepresentation, it is not unreasonable for him to fear that you will misrepresent and fool him in the business itself.

Advertisements by mail, reach the prospective customer at a time when he does not want them and they are usually put where he cannot find them. Acres of swampy land in cities are raised in level and made habitable by the contents of waste baskets. An advertisement in a journal is convenient to the customer, in time and place. Many physicians look over the advertisements as thoroughly as they do the technical contents of their journals.

Most of them study the advertisements for practical hints two or three times a year.

The manufacturer or merchant who advertises to his potential clientele in the periodicals of that clientele, is considering the convenience of that clientele. The man who advertises by dodgers and circulars is annoying the persons with whom he wants to do business. Dodger and circular advertising helps the printer and the collector of garbage, ashes and waste paper. Circular advertising perhaps helps Uncle Sam to maintain the postal service. Newspaper and magazine advertising helps the printer, the editorial worker of every grade, the publishing house, the reader of all kinds of current literature, indirectly the library, school and university. It makes possible, at low cost, a very important educational process among all classes of the community.

We want our readers to appreciate that every advertiser in this JOURNAL is helping to cut down the subscription price below what it could possibly be otherwise and is helping the JOURNAL to maintain and improve its standards of quality and quantity of reading matter.

We ask our readers to show their recognition of this fact by letting advertisers know, so far as is possible, that we are actually delivering to potential customers, the messages that the JOURNAL is paid to carry.

We also ask our readers for criticism in determining which of these messages are true and which, if any, are false. We cannot, off hand refuse to print an advertisement simply because of personal prejudice against a certain drug or instrument but we intend to limit our advertising pages to reputable and honest concerns.

---

SUMMER CASES.—Conditions peculiar to the season now with us will present themselves for your consideration and a reference to the fact that Antiphlogistine ha sproven of particular service in sunburn, bee sting, insect bites, sprains, bruises, and the like, will offer you a ready and satisfactory dressing and is procurable in all drug stores.

In those severe cases of dermatitis following undue exposure to the fact that Antiphlogistine has proven of particular service mation and the accompanying swelling and pain.

In all cases it should be applied thick and hot and well protected by ample covering.

